# Performance-related physiological changes induced by one year of endurance training in young athletes

**DOI:** 10.3389/fspor.2023.1149968

**Published:** 2023-05-10

**Authors:** Christina Mishica, Heikki Kyröläinen, Maarit Valtonen, Hans-Christer Holmberg, Vesa Linnamo

**Affiliations:** ^1^ Sports Technology Unit Vuokatti, Faculty of Sport and Health Sciences, University of Jyväskylä, Vuokatti, Finland; ^2^ Department of Sport and Health Sciences, University of Jyväskylä, Jyväskylä, Finland; ^3^ Research Institute for Olympic Sports (KIHU), Jyväskylä, Finland; ^4^ Department of Health Science, Luleå University of Technology, Luleå, Sweden

**Keywords:** VO_2_max (maximal oxygen uptake), cross-country skiing, adolescent & youth, sport-specific, double poling performance

## Abstract

**Introduction:**

Although maximal oxygen uptake (VO_2_max) is generally recognized as the single best indicator of aerobic fitness in youth, interpretation of this parameter and the extent to which it can be improved by training remain controversial, as does the relative importance of VO_2_max for performance in comparison to other factors such as power production. Here, we examined the influence of endurance training on the VO_2_max, muscle power and sports-related performance of cross-country skiers attending a school specializing in sports, as well as potential relationships between any changes observed to one another and/or to perceived stress scale (Cohen) and certain blood parameters.

**Methods:**

On two separate occasions, prior to the competition season and separated by one year of endurance training, the 12 participants (5 males, 7 females, 17 ± 1 years) carried out tests for VO_2_max on a treadmill, explosive power utilizing countermovement jumps (CMJ) and ski-specific maximal double pole performance (DPP) employing roller skis on a treadmill. Blood levels of ferritin (Fer), vitamin D (VitD) and hemoglobin (Hg) were monitored, and stress assessed with a questionnaire.

**Results:**

DPP improved by 10 ± 8% (*P* < 0.001), but no other significant changes were observed. There were no significant correlations between the changes in DPP and any other variable.

**Discussion:**

Whereas one year of endurance training improved the cross-country ski-specific performance of young athletes significantly, the increase in their maximal oxygen uptake was minimal. Since DPP was not correlated with VO_2_max, jumping power or the levels of certain blood parameters, the improvement observed probably reflected better upper-body performance.

## Introduction

Maximal oxygen uptake (VO_2_max) has been suggested to be the best single measure of performance (1). Thus, it has been studied for more than a century, yet the interpretation and trainability of VO_2_max is still a topic of controversy, especially in adolescents (2). Previous literature, concentrating on longitudinal development in young cross-country (XC) skiers, reveals mixed results. Research on athletes between 15 and 20 years of age, indicates that VO_2_max continued to increase, and high-level skiers continued to improve absolute VO_2_max values even after 20 years of age (3). Conversely, when adjusted for body weight maximal physiological parameters displayed minimal to no change in skiers from 17 to 18 years of age (4) with additional findings suggesting that males reach a VO_2_max plateau around 19-years of age (5). In junior XC skiers, the effect of endurance training is often evaluated on the basis of changes in VO_2_max (5, 6), although many other factors appear to be involved as well.

Previous research on juniors (18 years of age) found that roller-skiing performance in both diagonal-stride and double poling technique were accurate predictors for both male and females and numerous studies have shown that upper body power plays a major role in XC ski performance (7–10). With regards to upper-body muscular endurance training, as little as 6 weeks in well-trained adult skiers, showed improved double pole performance (7). These findings indicate that upper-body endurance and power are important determinants of XC performance, as well as that one year of ski-specific training should improve these parameters. Therefore, performance-related variables, such as the results of incremental double poling tests, would be of value to assess in connection with the development of young XC skiers.

Although it is now clear that the aerobic capacity of young athletes can be improved by training (2), the most appropriate method for monitoring and optimizing this training remains to be determined. Therefore, a variety of psychological, physiological, performance-related and biochemical measures are utilized to assess the effects of training (11–13). Psychological aspects are often evaluated on the basis of self-reported measures of perceived stress or mood, whereas physiological parameters are often quantified on the basis of performance, e.g., in submaximal or maximal exercise tests (12). Furthermore, biochemical and hematological measures help optimize an individualized balance between training and recovery, as well as reveal any potential nutritional deficiencies, which are common among athletes (14). Many athletes monitor their progress with a combination of these various markers (13), but it remains unknown which of them provide(s) the most validated and useful indicator(s) of performance (14), especially for young athletes.

The current investigation examined the potential influence of one year of endurance training on the results of performance-related tests and on other parameters commonly utilized to monitor the training of young XC skiers, in the present case students at a high school specializing in sports. Particular emphasis was placed on determining VO_2_max, fractional oxygen utilization at the timepoint at which the lactate threshold was reached, muscular power and double poling performance. Furthermore, we assessed potential relationships between these different performance-related measures, as well as between these and perceived stress and/or hematological parameters.

## Materials and methods

### Subjects

**Figure 1:**
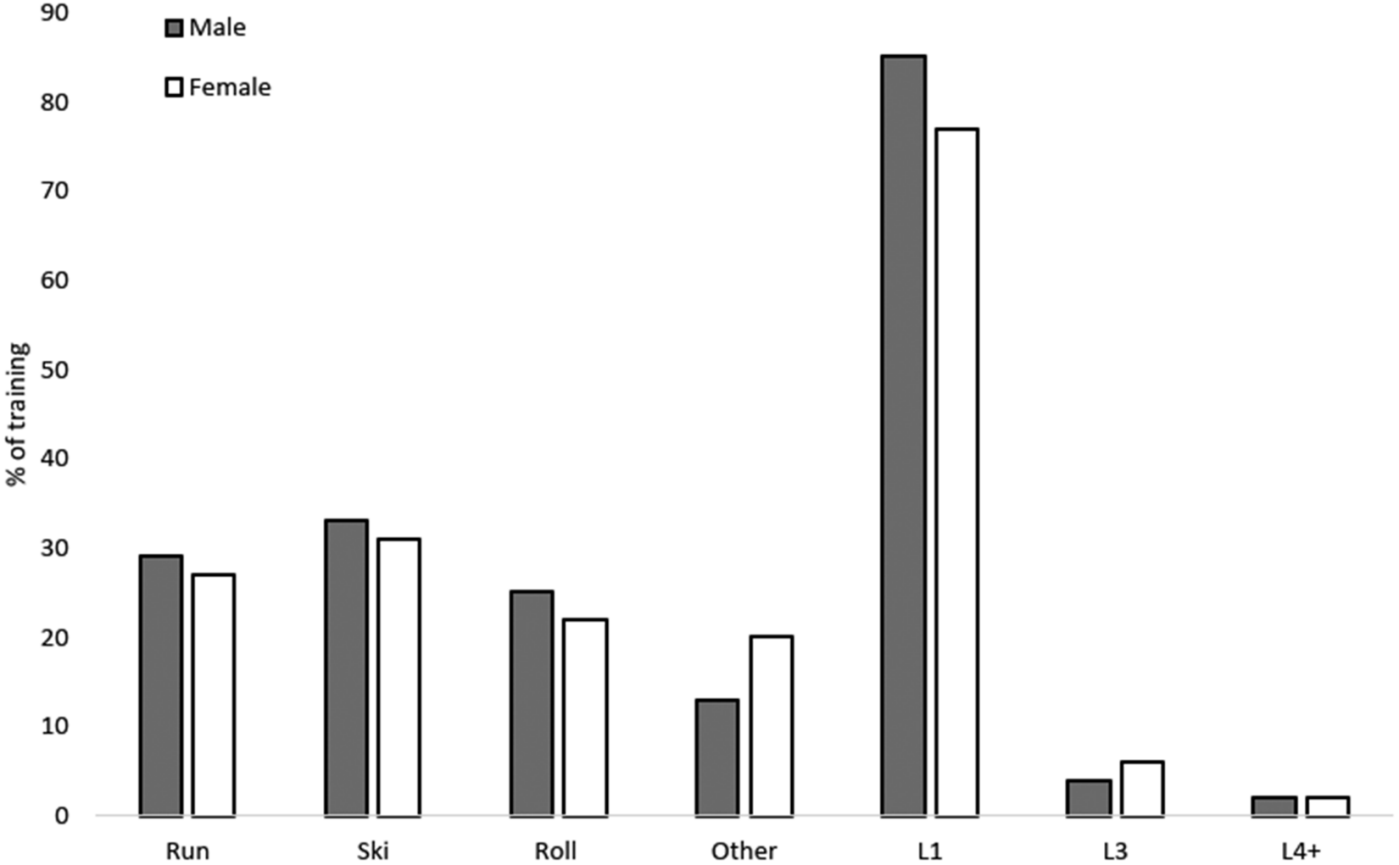
The modes of training and distribution of training intensity (TID) by young male and female cross-country skiers (endurance athletes) during a one-year period.

Twelve well-trained young endurance athletes who trained and competed in XC ski (9 subjects) and biathlon (3 subjects) year-round participated in this study. All subjects were attending a sports high school and had a minimum of 3 years of competition experience at the national level and can be classified as tier 4/tier 3 athletes according to McKay et al. 2022 (15). Baseline characteristics and training background are shown in Table 1 and details on gender specific training modes as well as training intensity distribution during the 12-month research period are shown in Figure 1. Subjects were fully informed of all the experimental procedures and provided written consent to participate in the study. The ethics committee of the University of Jyväskylä, Finland, approved the study, and measurements were performed in accordance with the declaration of Helsinki.

**Table 1:** Characteristics of subjects (means ± SD).

	Males (*N* = 5)	Females (*N* = 7)
Height (cm)	179.6 ± 5.4	169.2 ± 7.6
Body mass (kg)	72.4 ± 9.7	63.2 ± 6.3
VO_2_max (ml/kg/min)	68.4 ± 4.3	54.3 ± 4.3
Yearly training (hrs)	587 ± 35	562 ± 53

### Study protocol

The study was carried out during a one-year period that began and ended in November before the winter competition season began. Subjects were instructed to train according to their own individual training plans and report their training *via* electronic training diaries. In order to evaluate changes in physiological, psychological and performance-related variables, subjects were tested on two different occasions with one year time between tests. Subjects were administered the perceived stress survey (PSS) followed by assessment of explosive power with countermovement jumps (CMJ) and then a VO_2_max test to measure maximal oxygen consumption. Additional assessment included a ski-specific double pole test (DPT) on a roller ski treadmill and the monitoring of three commonly followed biomarkers (Fer, Vit D and Hg) from blood. Testing procedures were the same for all testing sessions, and subjects were instructed to do light training for 24 h prior to maximal performance tests. Jump exercises were already incorporated into the tested individuals' normal training so that subjects previously performed maximal tests a minimum of 2 times. Thus, a preparatory training period was unnecessary. Due to the longitudinal design of this study, measurement weeks were carefully considered with coaches to avoid weeks that included training camps, or exams and tests occurred during a 2-week period.

### Maximal oxygen uptake (VO_2_max)

Subjects completed a continuous, incremental, maximal test by walking or running with poles on a large motor-driven treadmill (Telineyhtymä, Kotka, Finland). The test started at an inclination of 2.8° with a speed of 5.5 km·h^−1^ for men at an inclination of 3.5° and speed of 5.0 km·h^−1^ for women. The workload increased every three minutes so that the predicted oxygen uptake calculated using the equation by Balke & Ware (16) increased by 6 ml·kg^−1^·min^−1^ in every stage. All subjects were familiar with this testing protocol and the test was considered maximal when the subject demonstrated clear signs of maximal effort such as, unsteady gait and/or an inability to continue, despite strong verbal encouragement. To ensure safety was met, participants wore a harness that was attached to a rope which hung from a frame in the ceiling above the treadmill.

Before each test, volume, and gas calibration of the ergospirometer was performed and resting values of heart rate and blood lactate were recorded. Throughout the test, breathing gases were measured using a mixed chamber system (Medikro 919 Ergospirometer, Medikro Oy, Kuopio, Finland). VO_2_ and respiratory exchange ratio (RER) from the last 60 s of each stage, and the highest 60 s average (VO_2_max) were recorded. Time to exhaustion (TTEVO2) was defined as the total number of minutes the subject walk/ran on the treadmill during the maximal test. Heart rate was continuously monitored using a Polar H10 heart rate belt (Polar Electro Oy, Kempele, Finland) and the average heart rate from the last 60 s of each stage was recorded. During the last 10 s of each stage, fingertip blood lactate samples were collected into capillary tubes (20 µl) and placed in a 1-mL hemolyzing solution. Once the test was complete samples were immediately analyzed using Biosen C-line analyzer (EKF diagnostics, Barleben, Germany).

Lactate threshold (LT) was defined as the warm-up lactate value (i.e., measured after the first stage) + 1.5 mmol·L^−1^. This method is in accordance with and recommended (17) by previous research (18, 19). For further analysis the percentage of oxygen utilization (VO_2_) at the defined LT was calculated based off the highest VO_2_ value (VO_2_max) that occurred during the maximal test.

### Explosive power (CMJ)

Explosive power was evaluated using CMJ (20). Prior to arrival, subjects completed a 15-minute warm-up running at a self-selected submaximal speed. Subjects were then instructed to jump as high as possible while keeping their hands fixed to their hips, feet shoulder-width apart and bending their knees to a 90-degree angle. A total of three jumps were completed with about 1-minute of recovery between jumps. Jumps were performed on a force plate (HUR FP8, HUR Oy, Kokkola, Finland), and jump height was calculated from impulse (21) using Coachtech system (Vuokatti Sports Technology Unit, University of Jyväskylä, Finland). The highest jump was recorded as the current measure of performance. CMJ are a common (13) and valid test for measuring fatigue and explosive power (20).

### Ski-specific performance (DPP)

Ski-specific performance was evaluated using maximal double pole performance tests. Tests were performed on a roller ski treadmill (Rodby Innovation AB, Vänge, Sweden) with Marwe 800 XC roller skis (Marwe Oy, Hyvinkää, Finland) equipped with prolink bindings (Salomon Group, Annecy, France) and standard 6C6 wheels (Marwe Oy, Hyvinkää, Finland). Subjects were instructed to bring their own individual classic ski boots and poles. A customized tip specific for treadmill roller skiing was then placed onto the subject's ski poles. Following an individual self-selected warm-up outside the laboratory, subjects performed a 10-minute warm-up on the roller ski treadmill at a workload equal to the first stage of the test. Towards the end of the warmup, each subject performed two 12–15 s sprints at a workload equal to the fourth through sixth stage of the test. This ensured the subject was ready and verified that the poles and additional equipment was working adequately.

After the warm-up, athletes performed an incremental treadmill test in the double pole technique at an inclination of 2° with men starting at 13 km·h^−1^ and women starting at 10 km·h^−1^. Throughout the test, inclination was constant, and speed increased by one km·h^−1^ every minute until volitional exhaustion. Time to exhaustion (TTEDP) and heart rate (during each stage) was recorded. All subjects were familiar with this testing protocol and verbal encouragement was used to help individuals obtain their best effort. To ensure safety was met, participants wore a harness that was attached to a rope which hung from a frame in the ceiling above the treadmill.

### Perceived stress scale (PSS)

Levels of perceived stress were assessed using the 14-item Cohen Perceived Stress Scale (PSS). The PSS is a reliable and validated psychological tool that was developed to evaluate stress from the psychological perspective. It consists of seven negative and seven positive items with the negative element intended to assess the lack of control and the positive element focused on the individual's ability to cope with existing stressors (14). A five-point Likert-type scale, ranging from 0, “Never” to 4, “Very Often” was used to rate each item. Possible scores ranged from 0 to 56 with higher scores indicating higher levels of perceived stress. Previously, the PSS has shown significant differences between healthy and overtrained athletes (22).

Subjects filled out the PSS upon their arrival to the testing laboratory. Surveys were conducted prior to CMJ and VO_2_max tests to ensure subjects were well rested and able to properly answer all questions without any external influence.

### Training analysis

Individual training plans were followed throughout the testing period. All participants recorded training data in electronic training diaries (elogger.net, Espoo, Finland). The subjects were familiar with recording training electronically and had previously reported training in this manner for at least one year prior to this research. Daily training and competition were recorded throughout the study period and training was analyzed according to the electronic training diaries. Physiological monitoring of training occurred on a daily basis with individual HR monitors (additional monitoring of blood lactate occurred on occasion) and subjects used individual training zones that were calculated from maximal exercise tests to help control and guide the intensity of their training. Due to the longitudinal design of this study, variations in how training intensity was reported were present and therefore, training intensity distribution was separated into three different training intensities: low intensity training (LI, blood lactate <2.0 mmol/L), lactate threshold training (LTT, blood lactate <4.0 mmol/L) and high intensity training (HI, blood lactate <4.0 mmol/L). In addition, the three most frequent training modes were reported including: running, skiing and roller skiing (Figure 1).

### Blood variables

Morning fasted blood samples were obtained from the antecubital vein for the analyses of hemoglobin, ferritin, and serum total 25-hydroxyvitamin D, e.g., serum 25(OH)D. Blood for the hemoglobin analysis was drawn into EDTA tubes (Greiner-Bio-One GmbH, Kremsmünster, Austria) and immediately further analyzed with Sysmex XP300 analyzer (SysmexCo., Kobe, Japan). For serum ferritin, the blood was drawn into Vacuette gel serum tubes (Greiner-Bio-One GmbH, Kremsmünster, Austria) and centrifuged for 10 min with 3,600 rpm to collect serum, which was then frozen to −20°C for further analysis. The samples were analyzed with Siemens Immulite 2000 XPI analyzer (Siemens Healthcare Lianberis, United Kingdom), where the serum ferritin was determined by using immunometric chemiluminescence method. The sensitivity of the assay for ferritin was 0.4 µ/L and the precision (CV%) for the assay was 4.6%. The measurements of serum 25(OH)D were performed using electrochemiluminescence immunoassays (ECLIA).

### Statistical analysis

Data are expressed as mean ± SD and were examined for the assumption of normal distribution before analysis using a Shapiro–Wilk test. To determine the effects of one-year of endurance training, we used a linear mixed model (LMM) for each of investigated variables. The analysis of a LMM has been widely used in longitudinal data when repeated measures of the same subjects are taken over the study period. This allows the assessment of within-subject changes over time as well as between-subject differences. The mixed model included gender (males or female), test (test 1 or test 2), and interaction terms between treatment and time as fixed effects and subject as random effects. The magnitude of differences between tests were expressed as standardized mean differences (Cohen's d effect size, ES) with the equation (M2-M1)/SDpooled. Due to gender differences, descriptive analysis was used to evaluate male and female results separately. To determine the associations between variables Pearson's correlation coefficients were used. Due to the small sample size, only whole group correlations for relative change between tests were analyzed. All statistical analysis were performed using SPSS version 26 (IBM SPSS Statistics 26, IBM GmbH, Munich, Germany). Statistical significance was set as *P* < 0.05.

## Results

### Performance tests

**Figure 2:**
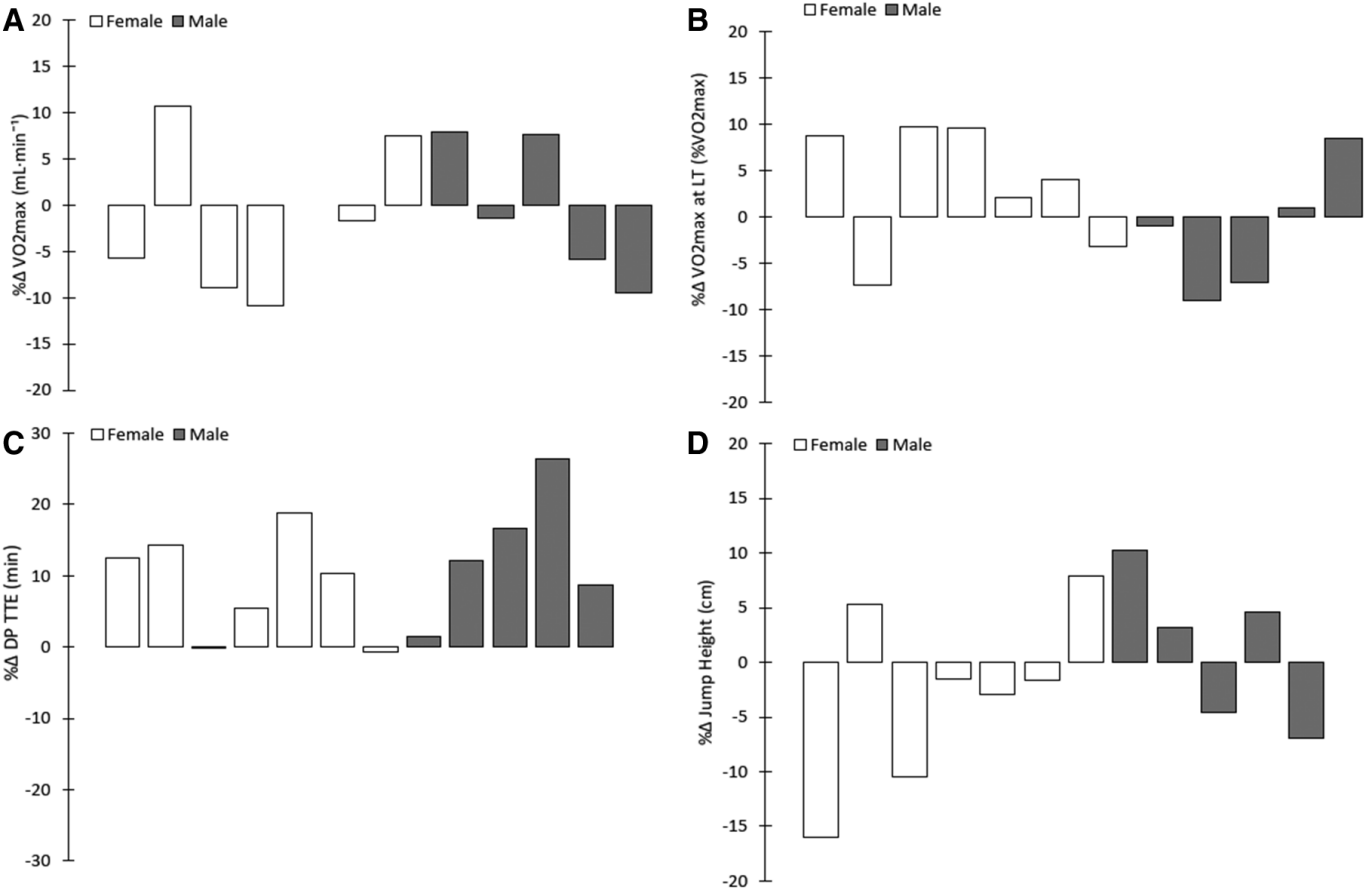
Individual changes (percentages) in (**A**) VO2max (ml/kg/min), (**B**) percentage of VO_2_max at the lactate threshold (LT), (**C**) duration of DP performance (DP TTE) and (**D**) counter-movement jump (CMJ) performance during the one-year of training.

Performance in the Double Poling Test (TTEDP) improved by 10 ± 8% (*P* < 0.001). Analysis of individual performances revealed that 10 of the young XC skiers improved, whereas two demonstrated a decline of 0%–1% (Figure 2). No significant correlations between DPP performance and any other variables were observed.

**Figure 3:**
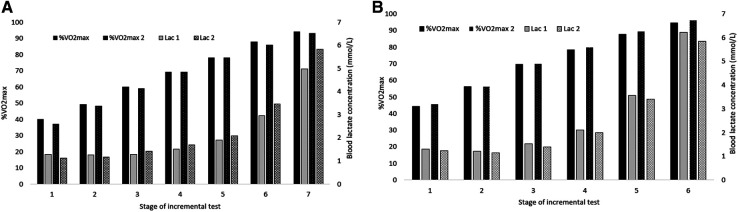
Changes in oxygen utilization and blood concentration of lactate during each individual stage of an incremental test following one year of endurance training in male (**A**) and female (**B**) cross-country skiers.

There was no significant change in of the other performance-related variables monitored. With respect to absolute VO_2_max, 7 of the athletes exhibited a decrease, 4 an increase and one no change (Figure 2). Table 2 documents the maximal physiological values in connection with each test and Figure 3 these values during each individual stage of the maximal incremental treadmill test, in both cases for both the men and women. A strong correlation was found between the changes in CMJ performance and absolute (r = 0.669, *P* < 0.05) and relative VO_2_max (r = .598, *P* < 0.05).

**Table 2:** Changes in performance-related variables, blood parameters and perceived stress in male and female cross-country skiers after 1-year of endurance training.

	Males, *n* = 5				Females, *n* = 7			
	Test 1	Test 2	% Change	Cohen's D	Test 1	Test 2	% Change	Cohen's D
VO_2_max								
ml·kg^−1^·min^−1^**	68.4 ± 4.3	68.0 ± 2.7	−0.2 ± 7.8	0.11	54.3 ± 4.3	53.4 ± 4.3	−1.3 ± 8.1	0.11
L·min^−1^**	4.9 ± 0.6	5.1 ± 0.7	3.2 ± 6.7	0.24	3.4 ± 0.3	3.4 ± 0.3	0.3 ± 8.3	0.24
TTE (min)**	24.8 ± 2.2	24.9 ± 1.1	0.6 ± 5.9	0.03	20.4 ± 1.2	19.9 ± 1.1	−2.0 ± 8.6	0.03
LT								
% VO_2_max	85.4 ± 7.4	83.9 ± 6.8	−1.5 ± 6.9	0.21	80.8 ± 4.7	83.4 ± 5.1	3.4 ± 6.7	0.21
Time LT (min)	17.2 ± 1.0	16.8 ± 2.0	−2.4 ± 8.9	0.25	13.2 ± 1.1	13.4 ± 1.1	2.0 ± 5.2	0.18
DP								
TTE (min)*	8.5 ± 1.8	9.5 ± 1.4	13.1 ± 9.3	0.61	8.0 ± 0.9	8.6 ± 0.9	8.6 ± 7.4	0.61
HRmax	195.8 ± 8.5	194.2 ± 7.2	−0.8 ± 1.5	0.21	197.3 ± 8.4	195.1 ± 6.7	−1.0 ± 2.0	0.20
CMJ								
Height (cm)**	36.6 ± 4.2	37.1 ± 4.9	1.3 ± 7.0	0.10	30.6 ± 4.2	29.8 ± 5.0	−2.8 ± 8.4	0.10
Blood parameter								
Ferritin**	68.5 ± 15.8	68.3 ± 19.9	−0.7 ± 36.4	0.75	47.9 ± 26.9	38.0 ± 17.3	−5.4 ± 72.2	0.75
Hemoglobin (g/L)**	150.8 ± 5.5	155.2 ± 4.2	1.9 ± 3.8	7.1	143.3 ± 7.3	144.9 ± 9.1	1.1 ± 4.4	7.21
Vitamin D (nmol/L)*	68.8 ± 35.9	82.2 ± 39.4	11.5 ± 19.2	0.72	81.4 ± 20.0	97.5 ± 25.1	4.5 ± 52.7	0.72
Perceived stress								
PSS (score)	12.6 ± 6.0	19.6 ± 12.5	66.7 ± 81.0	0.72	21.4 ± 9.4	23.9 ± 11.5	12.7 ± 56.2	0.72

Values are means and standard deviation (mean ± SD).

VO_2_max, maximal oxygen uptake; TTE, Time to exhaustion during test; LT, lactate threshold; DP, double pole test; HRmax, maximal heart rate during the double pole test; CMJ, countermovement jump test; PSS. Perceived Stress Survey.

* Significant effect of time (*P* < 0.05) on the linear mixed model (LMM) for each sex.

** Significant difference in LMM (*P* < 0.05) between the males and females.

### Perceived stress levels and training

As shown in Table 2, levels of perceived stress increased throughout the study period but individual variation was high. During the one-year period monitored, 85 ± 3% (458 ± 58 h) of the training by the men and 77 ± 10% (455 ± 80 h) by the women was LI. The distribution of HI training was similar for the men (2 ± 2%, 12 ± 9 h) and women (also 2 ± 1%, 10 ± 5 h), as was also the case for LTT training (4 ± 1%, 21 ± 10 h, and 6 ± 2%, 37 ± 14 h respectively). In addition, skiing was the mode of training utilized most (for the men, 33 ± 13%, and the women, 31 ± 7%) (Figure 1).

### Blood values

Neither Hg nor Fer significantly changed during the one-year training period, whereas the blood level of vitamin D increased in both the men and women (*P* = 0.042, Table 2).

## Discussion

The major finding here was that one year of training by young XC skiers improved their performance in ski-specific DP tests without altering any other significant performance-related parameter. In addition, although there was a slight increase in blood levels of vitamin D, the blood levels of Hg and Fer did not change. Nor was there any difference in perceived stress. Sex difference in physiological parameters was as expected, but no sex differences in the development of the different parameters were found over the one-year training period. A positive association was found between explosive power (CMJ) and VO_2_max (both absolute and relative).

### Performance-related variables

The increase in DP performance observed in this study indicates that during this one-year period subjects developed their upper-body performance. Previous research suggests that incremental DP tests appear to be a good predictor of XC skiers' performance (8) suggesting these improvements likely translated to competition results. It is interesting to note that the present improvement in the DP performance occurred without implementing a DP specific training intervention. This 10% improvement in TTEDP is considerably higher than previous research that reported a 3% increase in DP performance during 6 months of training (23). The current increase is comparable to previous studies that included a 6-week period of high intensity DP intervals and found 19.5% (9) and 16% (24) increases in performance. In the present study, anaerobic capacity during DP performance was not measured but maximal heart rate values during double pole tests were comparable to values obtained during VO_2_max tests (Table 2) suggesting that subjects were able to easily reach maximal levels in the DP technique. Due to the young age of the subjects, one may suggest that familiarization of treadmill skiing may contribute to the increase in performance. However, subjects were provided with 3–4 familiarization tests (each around 30 min) before the first test with an additional 2 sessions before the second test. Therefore, we considered that the skiers were well familiarized with treadmill DP technique and improvement is likely due to increased upper-body performance. This supports previous findings that reported maximal upper body strength having a substantial impact on DP roller skiing performance in both males and females (10). It should be noted, however, that time to exhaustion change in % cannot be directly compared to the underlying physiological capacity, or performance time change over a given distance. Previous studies have demonstrated that a 10% change in TTE might correspond to a change of approximately 1% in physiological capacity (25). In addition, the age of the current subjects is a factor to consider when interpreting results. Age-related differences (−10%) were found in time trial DP performance between 16 and 18-year-old vs. adult athletes suggesting that skiers at this age are still progressing, and their performance may benefit from additional training and/or further development of their neuromuscular system (23). Thus, the improvement in DP performance in the present study may be due to an additional training/development that occurred during the one-year study period.

Although athletes aim to improve VO_2_max values, a lack of change in VO_2_max despite adequate training commonly occurs. Earlier studies, also focused on young endurance athletes, showed no significant changes in VO_2_max during 3 years of endurance training (∼7 h week) (26) and no change in physiological and/or performance variables despite an increase in ski-specific training and volume for a period of 6 months (23). Additionally, one year of high-level training for 17-year-old male XC skiers showed minimal to no difference in absolute and relative physiological parameters at the maximal level and only a slight increase for females (4), further supporting the lack of change observed in the present study. However, it is important to note that the existing lack of differences was also influenced by high individual variation. The inter-individual variation in VO_2_max variables within our athlete cohort is most likely due to differences in trainability of VO_2_max, which is highly hereditary with age, sex, body composition and body mass (27). Figure 2 shows the individual changes in performance-related measures that were found between tests and interestingly, when looking at gender specific results, the oxygen consumption at LT increased for most of the female subjects. Previous research has shown an increase in oxygen consumption at similar thresholds in young male athletes but no change in females (4). However, in the present study this change was not significant and therefore, no conclusion can be drawn.

In terms of explosive power performance, CMJ results remained similar between tests in both males and females. However, when investigating the change between tests, a positive relationship was found between changes in jump height and changes in relative and absolute VO_2_max. Previous research has shown that 10-km run time (28) and TTE in middle-distance runners (29) was associated to vertical jump height suggesting that muscle power (neuromuscular performance) may be an important determinant of endurance abilities. This finding suggests the improvement in jump height may be reflected in maximal oxygen uptake, however, changes in jump performance in relation to VO_2_max have not been previously investigated in young skiers, therefore, the associations in the present study should be interpreted with caution.

A strength of the present study is the inclusion the of perceived stress and blood values variables. It is common practice for young athletes to monitor and follow blood values and therefore, improving knowledge and application of how to better interpret these values is important for coaches and athletes. For example, iron depletion (ferritin <12 µg/L, hemoglobin <13 µg/L for females and <30 µg/L for males), a prevalent nutrient deficiency among athletes, is frequently associated to an inadequate energy intake (30). Furthermore, young athletes have previously displayed a high rate of vitamin D insufficiency that further increased in the presence of iron depletion, suggesting that periodic screening of iron and vitamin D levels is important for young athletes (31). Few studies, assessing long-term changes in development and performance include practical applications and any indication of how these measures may aid in performance would be of high value to young athletes working to reach success on the elite stage.

As previously stated, no significant associations or changes were found between PSS and performance variables in the present study. This agrees with previous research following junior elite athletes of a similar age that showed minimal change during a 12-week intervention that was aimed at reducing PSS (32). The specific population of young elite athletes may be one possible explanation of this result since sport high schools often provide several resources and competent coaches/trainers that help balance training, competition, and school schedules by organizing the weekly training with an individual's total stress in mind (e.g., reduced training during examination weeks). Moreover, prior research has revealed that highly trained athletes are well prepared for competition with psychological response and functional strength of legs remaining unchanged after two consecutive days of high intensity competition (33). Contrary to the present findings, improved perceived recovery and stress contributed to an improved performance during one year of endurance training of female cyclists (31). These differences may be due to the reduced number of PSS measures, proposing that levels of perceived stress may need an increase in frequency to better reflect changes in performance. Considerable research has shown that stress influences performance and stress levels are important factors to consider when interpreting performance results (11, 12, 34, 35). Consequently, further research following measures of individual perceptions of stress alongside performance would be beneficial to help determine what level of perceived stress is tolerable for young athletes.

In this study, three blood parameters (Vit D, Hg and Fer) that are frequently followed by endurance athletes were analyzed. Although a variety of vitamin and minerals support the physiological processes that underline performance, evidence shows that nutritional deficiencies commonly occur in athletes, particularly for vitamin D and iron (14). In addition, living in a place where exposure to natural sunlight is limited (e.g., Finland during the winter months) amplifies the risk for vitamin D deficiency (36). Research following young Finnish runners reported vitamin D deficiency in approximately 68% of the subjects during the winter months (37) further demonstrating the importance of monitoring vitamin D status in young XC skiers. Recommended values of vitamin D based on bone health suggest 25(OH)D concentrations reach values >50 nmol · L^−1^ (38). In the present study vitamin D status was only monitored and no direction of supplementation was given but reference values were provided with 50–75 nmol · L^−1^ as a recommended range and a suggestion that athletes may want to be at levels <75 nmol · L^−1^ (39). In the present study, a significant increase occurred in 25(OH)D serum levels from test 1 (October 2019) to test 2 (October 2020). Although, detailed assessment of dietary vitamin D intake was not conducted, 75% of the participants reported taking vitamin D, iron, and other supplements irregularly throughout the current one-year training period. With regards to iron status, Fer and Hg both stayed at adequate levels with minimal to no change during the one-year period suggesting nutritional deficiencies or decreases in performance due to insufficient iron levels were not present. According to the findings from the present study, it appears that young XC skiers were able to maintain good metabolic and adequate nutritional profiles and vitamin D and/or iron status did not influence their performance.

As this study only had 12 subjects participating, there are some limitations due to the small sample size. This is a common issue in longitudinal research with high level athletes and this sample size is comparable to previous research following development and performance in XC skiers (5, 6, 8, 40). In addition, due to inconsistency in individual training diaries this study was unable to report strength training and training analysis was limited to three different intensities (LI, LTT and HI) and three different training modes. Furthermore, additional variables not measured in the current study, such as VO_2_max during double pole performance, ski-specific upper-body training and double pole economy should be included in future models to better understand what factors contribute to improved DP performance in young athletes.

## Conclusion

This study showed that young XC skiers improved ski-specific DP performance during one-year of sports high school, even though no change in maximal oxygen uptake, fractional oxygen utilization at LT, explosive power, perceived stress or selected blood biomarkers and endurance occurred. Although the improved DP performance did not directly influence the aerobic energy system (VO_2_max), we can predict that race performance was likely influenced by this significant change. Since the initial blood values were within the recommended range, a lack of change indicates that subjects maintained adequate dietary levels of vitamin D and iron during this one-year period. In addition, minimal changes in jump performance suggests that there were no major alterations in lower body capacity. Hence, the improved performance can, therefore, be explained by an improved upper-body performance. In addition, it appears analysis of one-year of endurance training in young skiers may require tests that include a ski-specific component and that other assessments of performance may not uncover valuable improvements that may motivate and provide young athletes with valuable insight needed for their future athletic careers.

## Data Availability

The datasets presented in this article are not readily available because ethical restrictions were placed upon this data due to the sensitive subject information and possible identifying information it may contain. In the informed consent form that was approved by the Research Ethical Committee we have stated that all data is confidential, and it will not be given to third parties. Our data includes sensitive health data and since all the subjects participating in this study were adolescents it is possible that they can be identified even after anonymization of this data, therefore, our data cannot be shared publicly. Requests to access the datasets should be directed to christina.m.mishica@jyu.fi, or to professor vesa.linnamo@jyu.fi, or to Secretary of the University of Jyväskylä Ethical Committee at secretaryethicomm@jyu.fi.
